# Anapole Modes in Hollow Nanocuboid Dielectric Metasurfaces for Refractometric Sensing

**DOI:** 10.3390/nano9010030

**Published:** 2018-12-27

**Authors:** José Francisco Algorri, Dimitrios C. Zografopoulos, Antonio Ferraro, Braulio García-Cámara, Ricardo Vergaz, Romeo Beccherelli, José Manuel Sánchez-Pena

**Affiliations:** 1 GDAF-UC3M, Displays and Photonics Applications Group, Department of Electronic Technology, Carlos III University of Madrid, Leganés, 28911 Madrid, Spain; brgarcia@ing.uc3m.es (B.G.-C.); rvergaz@ing.uc3m.es (R.V.); jmpena@ing.uc3m.es (J.M.S.-P.); 2 Consiglio Nazionale delle Ricerche, Istituto per la Microelettronica e Microsistemi, 00133 Rome, Italy; dimitrios.zografopoulos@artov.imm.cnr.it (D.C.Z.); antonio.ferraro@artov.imm.cnr.it (A.F.); romeo.beccherelli@artov.imm.cnr.it (R.B.)

**Keywords:** dielectric nanoparticles, anapole mode, metasurfaces, sensing devices

## Abstract

This work proposes the use of the refractive index sensitivity of non-radiating anapole modes of high-refractive-index nanoparticles arranged in planar metasurfaces as a novel sensing principle. The spectral position of anapole modes excited in hollow silicon nanocuboids is first investigated as a function of the nanocuboid geometry. Then, nanostructured metasurfaces of periodic arrays of nanocuboids on a glass substrate are designed. The metasurface parameters are properly selected such that a resonance with ultrahigh *Q*-factor, above one million, is excited at the target infrared wavelength of 1.55 µm. The anapole-induced resonant wavelength depends on the refractive index of the analyte superstratum, exhibiting a sensitivity of up to 180 nm/RIU. Such values, combined with the ultrahigh *Q*-factor, allow for refractometric sensing with very low detection limits in a broad range of refractive indices. Besides the sensing applications, the proposed device can also open new venues in other research fields, such as non-linear optics, optical switches, and optical communications.

## 1. Introduction

The field of silicon photonics has been in the forefront of scientific research in the last years as a promising platform to resolve the critical bottleneck in current microchip data transfer and consumption [1]. Taking advantage of the progress in fabrication techniques, the integration of photonic devices in close proximity to electronic circuits has been demonstrated [2], as well as the development of numerous components and devices, such as waveguides [3], lasers [4], or integrated sensors [5,6]. In parallel, significant attention has been directed to the study of the scattering properties of individual silicon nanoparticles [7,8,9], as potential building blocks in components compatible with silicon photonics technology. Silicon nanoparticles may support electric dipole resonances, which are very similar to plasmonic ones in metallic nanoparticles [10,11], with the advantage of avoiding thermal losses. Furthermore, magnetic resonances are also observed, which are relevant for several applications [12,13]. The scattering properties of such resonant modes can be tuned by properly designing the nanoparticle size and shape [14,15].

When various multipolar modes exist in the same structure, their coupling can lead to interesting interference effects. In this context, the control of the scattering directionality, following the work of Kerker et al. [16], was experimentally demonstrated in the microwave [17] and visible [7] spectra. By exploiting this concept, several applications have been proposed, e.g., waveguides [18], optical nanoswitches [19], dielectric mirrors [20], and sensors [21]. Recently, interference phenomena generating anapole modes have been experimentally demonstrated in silicon nanodisks [22]. Anapole modes stem from the destructive interference between an electric and toroidal dipole mode with opposite phase and identical angular distribution. Their main characteristic is that such modes are non-radiating and thus can potentially cloak the nanodisk at the anapole wavelength. Recent applications take advantage of such properties in nanolasers [23], beam steering [24], broadband absorption [25], ideal magnetic scattering [26], enhanced nonlinear effects [27,28] and high *Q*-factor devices [29,30].

In this work, we theoretically propose and demonstrate a refractometric sensor based on a metasurface of silicon nanocuboids supporting anapole modes. First, the tunability of the spectral position of the anapole mode in silicon nanocuboids is theoretically studied as a function of the nanoparticle thickness, aspect ratio, hole size and surrounding refractive index. Then, metasurfaces of properly selected hollow nanocuboids are designed, which lead to ultrahigh *Q*-factor resonances ($Q>10^{6}$) stemming from the interference between the anapole mode and the collective metasurface electromagnetic response. The metasurfaces are patterned on a standard glass substrate, where the superstratum volume is occupied by an analyte with unknown refractive index. The resonant wavelength strongly depends on the analyte index, showing sensitivity values of up to 180 nm/RIU in the target infrared telecom bands. More important, the ultrahigh *Q*-factor design, which leads to sub-picometric resonance linewidth, makes the proposed devices as ideal candidates for sensing with very low detection limits [31].

The paper is structured as follows: Section 2 describes the multipole decomposition method for the investigation of the scattering properties of the multipole modes in dielectric nanoparticles. Section 3 is divided in two subsections; the results on the scattering cross-sections of individual nanoparticles in Section 3.1, whereas the transmittance optical response of the designed dielectric metasurfaces are presented in Section 3.2. Finally, conclusions are summarized in Section 4.

## 2. Methods

The scattering properties of the dielectric nanocuboids are investigated by decomposing the fields inside the nanoparticles into Cartesian multipole moments. This widely employed method permits the calculation of the contributions stemming from toroidal moments and hence the identification of the conditions for the excitation of anapole modes [32,33,34,35,36]. Assuming the $e^{j\omega t}$ convention for the harmonic electromagnetic fields, the induced polarization current density J(r)

(1) $$J\left(r\right)=j\omega \left(\varepsilon_{p}-\varepsilon_{b}\right)E\left(r\right),$$

where E(r) is the electric field, ω is the angular frequency, and $\varepsilon_{p}=\varepsilon_{r,p}\varepsilon_{0}$ and $\varepsilon_{b}=\varepsilon_{r,b}\varepsilon_{0}$ are the permittivities of the nanocuboid and the background medium, respectively, while $\varepsilon_{0}$ is the vacuum permittivity. Both media are non-magnetic, therefore the relative permittivities $\varepsilon_{r,p}$, $\varepsilon_{r,b}$ are equal to the square of the corresponding refractive indices $n_{p}$, $n_{b}$ for the particle and the background medium. The electric field E(r) is calculated numerically by means of the finite-element frequency-domain technique implemented in the commercial software Comsol Multiphysics™.

The dipolar moments for the electric, magnetic, and toroidal modes are expressed as

(2) $$p=\frac{1}{j\omega }\int_{V}J\left(r\right)dr$$

(3) $$m=\frac{1}{2\upsilon_{b}}\int_{V}\left[r\times J(r)\right]dr$$

(4) $$t=\frac{1}{10\upsilon_{b}}\int_{V}\left[\left(r\times J(r)\right)r-2r^{2}J\left(r\right)\right]dr,$$

where $\upsilon_{b}=c/n_{b}$ is the speed of light in the surrounding medium and the integral is calculated in the nanoparticle volume. The electric, magnetic, and toroidal quadrupole moments are calculated as

(5) $$Q_{\alpha \beta }^{e}=\frac{1}{j2\omega }\int_{V}\left[r_{\alpha }J_{\beta }+r_{\beta }J_{\alpha }-\frac{2}{3}\delta_{\alpha \beta }\left(r\times J(r)\right)\right]dr$$

(6) $$Q_{\alpha \beta }^{m}=\frac{1}{3\upsilon_{b}}\int_{V}\left\{{\left[r\times J(r)\right]}_{\alpha }r_{\beta }+{\left[r\times J(r)\right]}_{\beta }r_{\alpha }\right\}dr$$

(7) $$\begin{array}{cc} Q_{\alpha \beta }^{T}=\frac{1}{28\upsilon_{b}}\int_{V} & [4r_{\alpha }r_{\beta }\left(r\times J(r)\right)\;-\;5r^{2}\left(r_{\alpha }J_{\beta }\;+\;r_{\beta }J_{\alpha }\right)\\ & +2r^{2}\delta_{\alpha \beta }\left(r\times J(r)\right)]dr, \end{array}$$

where the subscripts α,β=x,y,z and δ is the Dirac delta function. We also include the mean-square radii corrections for the magnetic and toroidal dipole, given by

(8) $${\bar{R}}_{m}^{2}=\frac{1}{2\upsilon_{b}}\int_{V}\left[r\times J(r)\right]r^{2}dr,$$

(9) $${\bar{R}}_{t}^{2}=\frac{1}{28\upsilon_{b}}\int_{V}\left[3r^{2}J\left(r\right)-2r\left(r\times J(r)\right)\right]r^{2}dr.$$

The mean-square radius correction for the electric dipole is omitted, since it does not contribute to the far-field radiation [32].

The scattering cross-sections corresponding to each multipole moment are given by

(10) $$C_{\mathrm{sca}}^{\left(\mathrm{ed}\right)}=\frac{k_{b}^{4}}{6\pi \varepsilon_{b}^{2}}{\left|p-jk_{b}\left(t+\frac{k_{b}^{2}}{10}{\bar{R}}_{t}^{2}\right)\right|}^{2},$$

(11) $$C_{\mathrm{sca}}^{\left(\mathrm{md}\right)}=\frac{k_{b}^{4}}{6\pi \varepsilon_{b}^{2}}{\left|m-\frac{k_{b}^{2}}{10}{\bar{R}}_{m}^{2}\right|}^{2}$$

(12) $$C_{\mathrm{sca}}^{\left(\mathrm{eq}\right)}=\frac{k_{b}^{6}}{20\pi \varepsilon_{b}^{2}}\sum_{\alpha \beta }{\left|Q_{\alpha \beta }^{e}-j\frac{k_{b}}{3}Q_{\alpha \beta }^{T}\right|}^{2}$$

(13) $$C_{\mathrm{sca}}^{\left(\mathrm{mq}\right)}=\frac{k_{b}^{6}}{80\pi \varepsilon_{b}^{2}}\sum_{\alpha \beta }{\left|Q_{\alpha \beta }^{m}\right|}^{2},$$

where an incident planewave with electric field amplitude $\left|E_{0}\right|=1$ V/m is assumed, $k_{b}=2\pi n_{b}/\lambda$ is the wavevector in the surrounding medium, and the superscripts (ed), (md), (eq), (mq) denote the electric dipole, magnetic dipole, electric quadrupole, and magnetic quadrupole, respectively.

When the anapole modes are sought, it is convenient to define the magnitudes $C_{\mathrm{sca}}^{p}$ and $C_{\mathrm{sca}}^{t}$

(14) $$C_{\mathrm{sca}}^{p}=\frac{k_{b}^{4}}{6\pi \varepsilon_{b}^{2}}{\left|p\right|}^{2}$$

(15) $$C_{\mathrm{sca}}^{t}=\frac{k_{b}^{6}}{6\pi \varepsilon_{b}^{2}}{\left|t+\frac{k_{b}^{2}}{10}{\bar{R}}_{t}^{2}\right|}^{2},$$

which correspond to the effective cross-sections of the Cartesian electric and toroidal dipoles, respectively. Depending on their relative phase difference, these two dipoles may interfere constructively or destructively and their combined contribution to the total electric dipole scattering cross-section is described in Equation (10). Nevertheless, the separate calculation of Equations (14) and (15) facilitates the design of the proposed structures and provides further insight on the excitation of the anapole modes, which occurs at a crossover of $C_{\mathrm{sca}}^{p}$ and $C_{\mathrm{sca}}^{t}$. It is interesting to note that the contribution of the Cartesian toroidal moment t to the total electric dipole cross-section $C_{\mathrm{sca}}^{\left(\mathrm{ed}\right)}$ depends on the surrounding medium permittivity via the factor $-jk_{b}/10\upsilon_{b}$ (cf. Equations (4) and (10)), contrary to the electric moment p (cf. Equation (2)). This implies that the anapole mode can be manipulated by changing the surrounding conditions [36], a property that is highly relevant in the context of refractometric sensing, which is investigated in this work.

## 3. Results

This section is organized in two subsections devoted to the study of single particles and periodic metasurface arrangements, respectively. In Section 3.1, the multipolar decomposition of solid and hollow silicon nanocuboids is performed, investigating the tunability by design of the anapole mode by modifying the nanocuboid geometry and the surrounding refractive index. Section 3.2 presents the optical response of metasurfaces, properly designed to achieve resonances with ultrahigh *Q*-factors, which stem from the excitation of anapole modes in the nanocuboids. Although various shapes have been investigated for the nanoparticle building blocks, such as disks, hollow disks, solid and hollow-core nanocuboids, it has been observed that hollow nanocuboids produce the highest *Q*-factors, so the analysis is focused on this geometry.

### 3.1. Multipole Decomposition of Individual Nanoparticles

As any other effect related to Mie resonances, the excitation of anapole modes depends on the particle size and aspect ratio. These modes are supported only within a certain range of aspect ratio values. Furthermore, the observance of the anapole mode in isolated nanoparticles requires a negligible contribution of other higher-order multipole moments. For instance, aspect ratios between 0.125 and 0.25 are required in order to observe the anapole mode in dielectric nanodisks [22]. In rectangular nanoparticles, an aspect ratio around 0.2 leads to an anapole mode with all other multipolar moments almost suppressed [25]. In this work, we demonstrate how the aspect ratio, hole size and surrounding refractive index can be used to tune the multipole moment contributions, focusing on the anapole mode. Neglecting material dispersion, dimensions can be scaled such that the resonant wavelengths lie in the near-infrared spectrum, where measurements are straightforward thanks to the available techniques used in optical communication systems.

**Figure 1:**
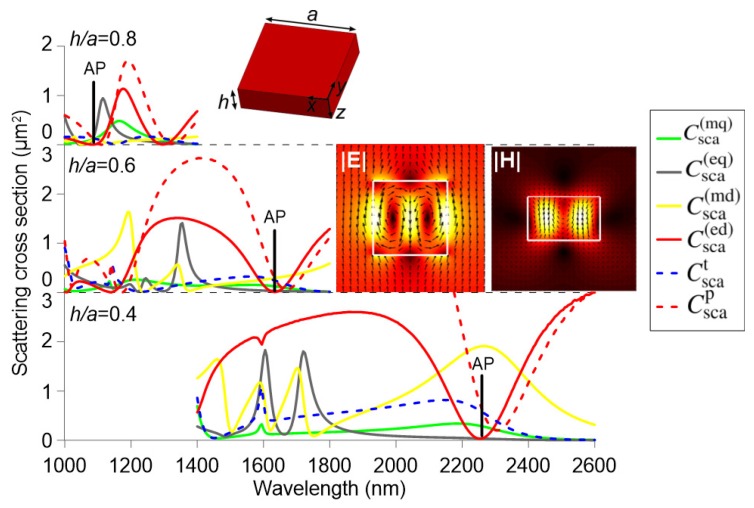
Multipole decomposition for a solid silicon cuboid nanoparticle (h=361 nm) for three different aspect ratios h/a=0.4, 0.6, and 0.8. The surrounding medium is air. The anapole wavelength in each case is marked as “AP”. The insets show the electric and magnetic field profiles at the x−y and x−z planes, respectively, calculated at the anapole wavelength of 1635 nm for h/a=0.6.

Figure 1 shows the scattering cross-sections of the dipole and quadrupole moments (as defined in Section 2) for a solid silicon nanocuboid with thickness h=361 nm and a square cross-section of size *a*, for different values of the aspect ratio h/a. The impinging planewave is y-polarized and propagates along the *z*-axis, as defined in the inset of Figure 1. The refractive index of silicon and its dispersion in the considered wavelength range is taken into account according to the experimental values reported in [37], whereas air is considered as the surrounding medium. In the three cases examined, a minimum of the total electric dipole scattering cross-section is observed at the crossing point between the Cartesian electric and toroidal moments, where they interfere destructively. That wavelength corresponds to the anapole mode, indicated by the label “AP”. The electric and magnetic field profiles shown in the inset were calculated at the anapole wavelength of 1635 nm for h/a=0.6 and they exhibit loops of displacement current and circular magnetic moment, characteristics of the anapole mode [22,30,38,39].

The bottom graph of Figure 1 shows the scattering cross sections of the multipole moments for a nanocuboid of aspect ratio h/a=0.4 (h=361 nm, a=902 nm). As it can be observed, the magnetic dipole is very strong at the anapole wavelength, which hinders the observation of the anapole mode in isolated nanoparticles. There is an important contribution to the total scattering of the electric quadrupole at shorter wavelengths, far from the anapole mode, whereas the magnetic quadrupole is almost negligible. For a ratio of h/a=0.6 (h=361 nm, a=602 nm), the multipolar contributions shift to shorter wavelengths, as the volume of the nanoparticle shrinks. The anapole mode shifts from 2265 to 1630 nm (∆λ=635 nm), getting closer to the target infrared wavelength of 1.55 µm, while the electric quadrupole peak shifts from 1730 to 1355 nm (∆λ=375 nm). Thus, the anapole mode wavelength approaches that of the electric quadrupole, as it experiences an almost double shift. For an aspect ratio of h/a=0.8 (h=361 nm, a=451 nm), the anapole mode further shifts to a wavelength lower than that of the electric quadrupole. This behavior can be used to arbitrarily tune the anapole mode, shifting it closer or further from other multipolar contributions.

**Figure 2:**
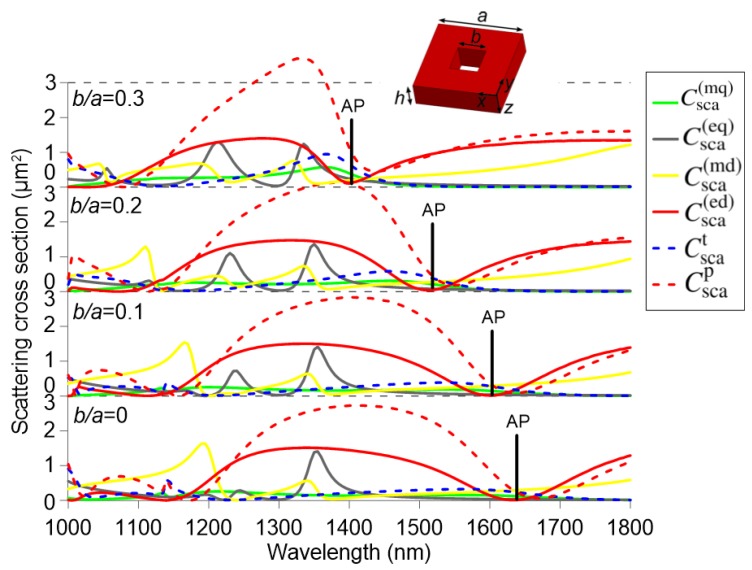
Multipole decomposition for a silicon cuboid nanoparticle (h=361 nm, h/a=0.6) for four different values of the central hole size: b/a=0, 0.1, 0.2, and 0.3. The surrounding medium is air.

Figure 2 takes as a starting point a value of h/a=0.6. Then, a central hole with a square cross-section of size *b*, is introduced in the nanocuboid, as shown in the inset of Figure 2. The relative size of the hole is modified from b/a=0 to b/a=0.3. For comparison, the bottom graph of Figure 2 replots the scattering cross sections for a solid nanocuboid of h/a=0.6 (h=361 nm, a=602 nm). In that case, the anapole mode wavelength is more than 250 nm larger than that of the next important contribution, which is the electric quadrupole. The introduction of a hole of b/a=0.1 shifts the anapole mode from 1630 to 1605 nm, while the wavelengths corresponding to the peaks for the other multipole contributions remain mostly unaffected. The same trend is observed for b/a=0.2, where a shift of the anapole mode of more more than 90 nm with respect to the solid nanocuboid is observed. Finally, for a hole of b/a=0.3, there is no longer a crossing between the electric and toroidal dipole, but rather a wavelength where their values are still close, leading to a minimum in the total electric dipole cross-section at 1405 nm, namely a total shift of 110 nm with respect to the reference case of the solid nanocuboid. These results demonstrate that the the anapole mode can be tuned in a large spectral range simply by modifying the hole size of the nanocuboid with the advantage that other multipolar contributions are almost unaffected.

**Figure 3:**
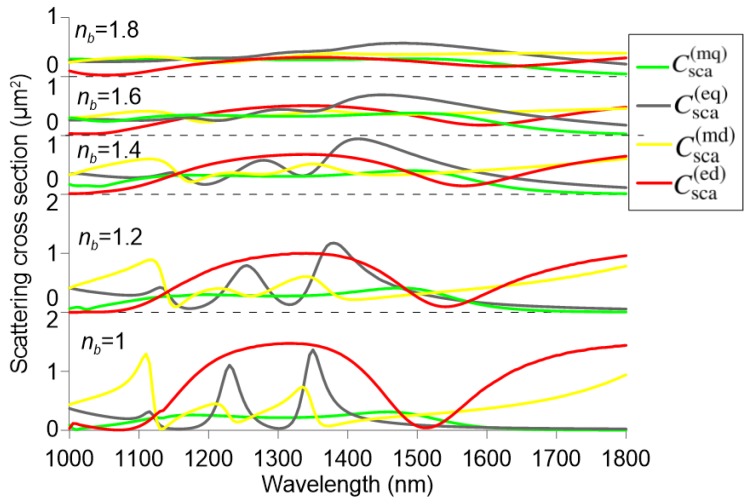
Multipole decomposition for a silicon cuboid nanoparticle (a=602 nm, h/a=0.6, b/a=0.2) for different values of the refractive index $n_{b}$ of the surrounding medium.

Finally, for sensing applications, the most relevant tuning mechanism is the refractive index $n_{b}$ of the surrounding medium. In this aspect, we have considered a broad range of refractive indices, from $n_{b}=1$ to 1.8. This range covers all possible analyte materials, such as air and gases (1–1.1), water and biosolutions (1.3–1.5), and various optical liquids (1.5–1.8). Figure 3 investigates the effect of the surrounding refractive index in the aforementioned range for a hollow nanocuboid with h/a=0.6, a=602 nm, h=361 nm, and b/a=0.2. It is observed that the minimum of the total electric dipole cross-section $C_{\mathrm{sca}}^{\left(\mathrm{ed}\right)}$ shifts towards higher wavelength for increasing $n_{b}$, which is the mechanism relevant for refractometric sensing. Moreover, higher values of $n_{b}$ lead to lower refractive index contrast between the silicon nanocuboid and the surrounding medium, which hinders the appearance of strong resonances and their observation due to the broadly overlapping spectra. Nevertheless, when the nanoparticles are arranged in a metasurface configuration, the radiative damping can be minimized due to collective oscillations mediated by near-field coupling between the unit cells [40,41], leading to a different scattering response. This approach is exploited in the next Subsection, where metasurfaces composed of silicon nanocuboids are investigated as refractometric sensors.

### 3.2. Nanostructured Metasurfaces

The control of the optical response is the most relevant feature in the design of novel nanostructured surfaces or metasurfaces. For instance, an ideal metasurface of dielectric particles supporting the anapole mode may exhibit zero/minimum transmittance, depending on the interference of the anapole mode with other modes supported by the metasurface [42,43]. Here, we demonstrate that by properly selecting the nanocuboid and metasurface geometry, it is possible to engineer metasurfaces with very high *Q*-factor resonances originating from the anapole mode in both operation modes, namely transmission and reflection. In the first case, a metasurface with a sharp electromagnetically induced transparency (EIT) response at 1.55 µm is designed. In the second, an ultrahigh *Q*-factor reflective notch filter is demonstrated at the same wavelength. In both cases, the sensitivity of the resonant wavelength on the superstratum analyte refractive index is investigated.

**Figure 4:**
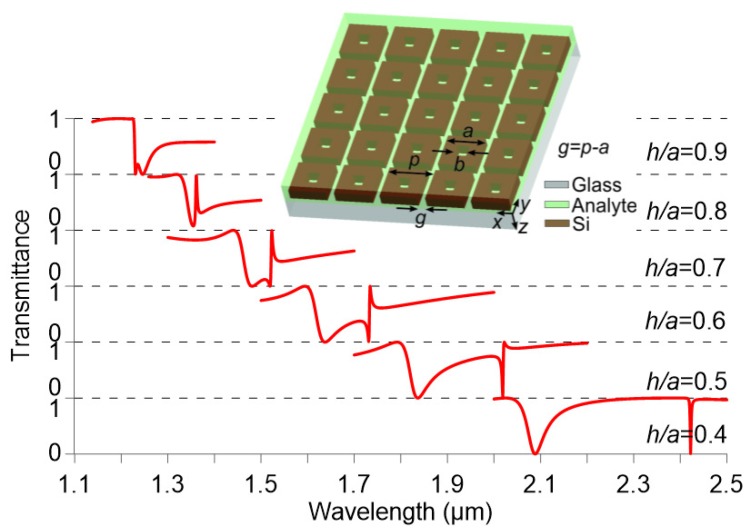
Transmittance of a metasurface composed by solid silicon nanocuboids (h=361 nm, b/a=0, g=50 nm) patterned on a glass substrate for different aspect ratios. The refractive index of the analyte is $n_{a}=1.5$.

The inset of Figure 4 shows the schematic of the investigated metasurfaces. A thin layer of silicon grown on a commercial glass substrate (Borofloat^®^ 33, characterized by an index of $n_{g}=1.456$ at 1.55 µm), is patterned so as to define the nanoparticle array. The superstratum is occupied by the volume of the analyte, which has an unknown refractive index $n_{a}$. The optical properties of the metasurface are investigated by means of the finite-element method (Comsol Multiphysics™) by defining appropriate periodic conditions at the *x*-*z* and *y*-*z* boundary planes of the unit cell. A *y*-polarized planewave impinges perpendicularly on the metasurface and the power transmittance of the structure is calculated. Due to the subwavelength pitch of the metasurface array, the diffraction of the incident beam is zero in the cases examined.

The effect of the metasurface pitch *p*, or equivalently the gap g=p−a, is a key parameter of the design. When the nanocuboids are arranged in a metasurface configuration their coupling can lead to very sharp resonances, facilitated by their rectangular shape which makes such coupling higher than among nanodisks, for instance. For this reason, in addition to the multipole resonances of the isolated nanocuboids, their strong radiative coupling affects the total response of the metasurface [30,40,41]. When the gap between cuboids is too large, the observation of the anapole mode is hindered by other multipole resonances, leading to a spectral response with broad features, not useful for a practical sensing device. It has been observed that a reduction on the gap increases the *Q*-factor of the target resonances and reaches a maximum value at a value of g=50 nm. Further shrinking of the gap complicates the fabrication of the metasurface without improving its optical response. For these reasons, in all studies presented here a gap of 50 nm has been considered.

Similar to the analysis for isolated nanoparticles in Figure 1, the effect of the nanocuboid aspect ratio on the metasurface response is investigated in Figure 4 for the case of solid nanocuboids. Their height is 361 nm, the gap is 50 nm, and the surrounding refractive index is 1.5, as a midpoint between 1 and 2 and a common refractive index of many optical liquids. The results of Figure 4 reveal the same behavior as that of Figure 1. The bottom graph of Figure 4 shows the transmittance of a metasurface with h/a=0.4 (h=361 nm, a=902 nm). Two main resonances are observed, a sharp resonance at higher wavelengths, associated with the anapole mode, and a second resonance at shorter wavelengths caused by the interference of other multipole modes. As the aspect ratio increases, the resonances shift to shorter wavelengths, although much faster in the case of the anapole mode resonance. This effect can be used to tune the spectral distance between resonances. Aspect ratios higher than 0.8 make the anapole mode to overlap with the broad low-transmittance resonance, as it was also demonstrated in Figure 1.

**Figure 5:**
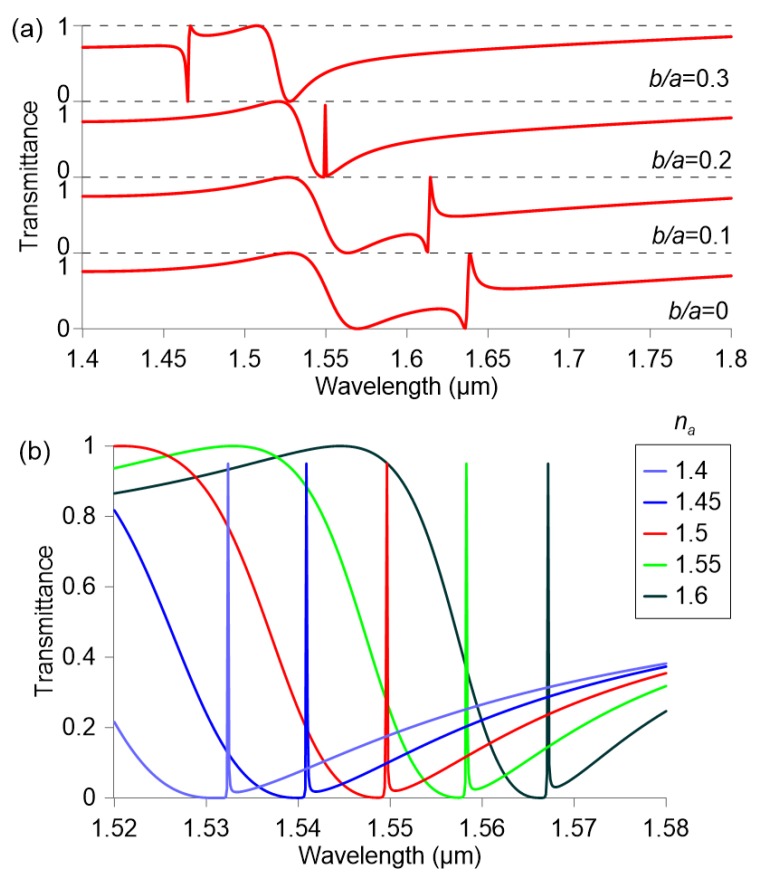
Transmittance of a metasurface composed by silicon nanocuboids (h=361 nm, h/a=0.64, g=50 nm) patterned on a glass substrate for (**a**) different relative hole size ($n_{a}=1.5$) and (**b**) different analyte refractive index (b/a=0.21).

In order to obtain the stronger EIT response at 1.55 µm, the two resonances have to be degenerate. This can be achieved by adjusting both the aspect ratio of the nanocuboid and the size of the hole. Figure 5a investigates the effect of the hole size for a fixed aspect ratio h/a=0.64. As already observed in Figure 2, modifying the hole size can tune the anapole mode Fano resonance wavelength from $\lambda_{r}=1.64$ to 1.46 µm, without significantly affecting the spectral background originating from other modes. Thus, by optimizing the geometry of the nanocuboid a sharp EIT response is observed at ∼1.55 µm, whose *Q*-factor is maximized for b/a=0.21.

Having selected the optimal geometrical parameters, the optical response of the metasurface is investigated as a function of the analyte refractive index. As demonstrated in Figure 3, a variation of the refractive index shifts the multipole moment resonant wavelengths and hence the EIT resonance of the metasurface. This effect can be used as a sensing mechanism, with a sensitivity $S=∆\lambda_{r}/∆n_{a}$ equal to 171 nm/RIU. The resonance linewidth $∆\lambda_{\text{3-dB}}$, defined as the spectral distance between the two wavelengths where the transmittance is half its peak value, obtains a minimum value of 60 pm, when the analyte index matches that of the substrate. The corresponding *Q*-factor, defined as $Q=\lambda_{r}/∆\lambda_{\text{3-dB}}$ equals $2.5\times 10^{4}$. Although this design provides significant sensitivity and narrow linewidths, the EIT response can manifest only within the spectral width of the low-transmittance background, which limits the span of the sensor approximately in the refractive index range from 1.5 to 1.6.

Given these considerations and taking advantage of the geometrical tunability of the anapole mode, the next design targets a flat maximum transmittance spectral window in which the anapole mode manifests as a selective dip in transmission. As a first step, the aspect ratio of the nanocuboid is chosen such that the anapole mode overlaps with the broad resonance manifested for a solid nanocuboid, as shown in Figure 4 for aspect ratios higher than 0.8. A value of h/a=0.88 is selected, which corresponds approximately to the upper graph of Figure 4. Since the resonance has to be shifted to longer wavelengths in order to match the target wavelength of 1.55 µm, the nanocuboid height is increased from 361 to 480 nm.

**Figure 6:**
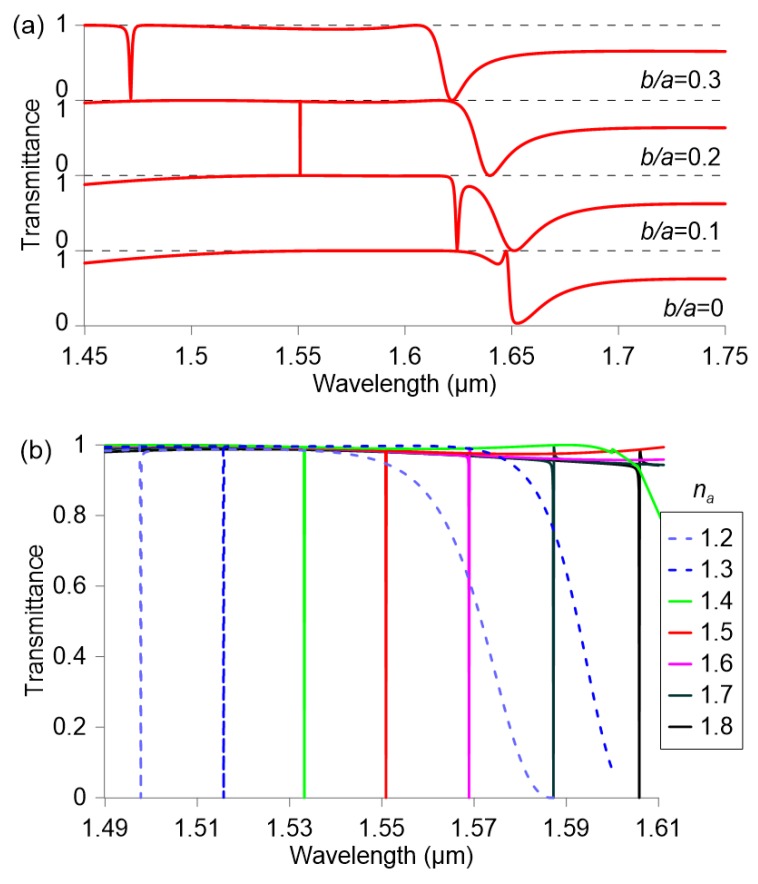
Transmittance of a metasurface composed by silicon nanocuboids (h=480 nm, h/a=0.88, g=50 nm) patterned on a glass substrate for (**a**) different relative hole size ($n_{a}=1.5$) and (**b**) different analyte refractive index (b/a=0.21).

Then, the effect of the hole size is investigated in the range b/a=0 to 0.3, with the results shown in Figure 6a for the following set of parameters: h/a=0.88, a=545 nm, h=480 nm, g=50 nm, and $n_{a}=1.5$. The increase of the hole size from b/a=0 to 0.3 shifts the resonance from 1.64 µm to 1.46 µm while the broadband low-transmittance resonance is only shifted from 1.65 µm to 1.63 µm. Therefore, this configuration permits the tuning of the sharp anapole resonance withing a large spectral window of almost 100% transmittance, isolated from other spectral features. The resulting interference results in narrowband reflection at the resonant wavelength [43], contrary to the EIT-like transmittance evidenced in Figure 5b. More importantly, the *Q*-factor is dramatically increased due to the weak coupling of the interfering modes, which leads to very high confinement of the electromagnetic field in the near-field zone. As in the previous design, the maximum *Q*-factor is achieved for a hole size of b/a=0.21, but its value is almost two orders of magnitude higher. In particular, the optical transmittance of the metasurface as a function of the analyte refractive index is investigated in Figure 6b. The obtained sensitivity is 180 nm/RIU and it remains almost constant in the whole span from $n_{a}=1.2$ to 1.8 thanks to the broad spectrum, flat, high-transmittance background. A minimum linewidth of 0.9 pm is calculated, which corresponds to an ultrahigh *Q*-factor value of $1.71\times 10^{6}$.

Finally, we have studied the effect of losses, both in the dielectric nanocuboids and the analyte material, by changing the imaginary part *k* of the refractive index of silicon and the analyte. In the case of silicon, the optical response is not affected up to values of $k=10^{-7}$, which exceed by far [44] or are within the range [37] of experimentally measured values for polycrystalline silicon. The losses of the analyte are a critical issue, as they may provide the ultimate resolution limit for the sensor, as discussed in [45]. It has been observed that for values up to $k=10^{-4}$ the quality factor of the resonance does not deteriorate, whereas a further increase increases both the resonance linewidth and the transmittance at the resonant wavelength. For extinction coefficients higher than $k=10^{-3}$ the resonance is lost.

There is an abundance of optical refractometric sensor techniques based on the interaction between the probe light beam and the analyte material based on a broad range of devices and components, among which optical fibers and waveguides, photonic crystals, planar resonant cavities, microresonators, metallic metamaterials and metasurfaces, and surface plasmon polariton prism and gratings [46,47,48,49]. Although in some of these approaches the sensitivity can exceed by more than one order of magnitude the values achieved by the proposed refractometric sensor, the latter features a series of advantages, which demonstrate its potential, namely: (i) sub-picometric linewidth, which combined with state-of-the-art optical spectrum analyzers and temperature stabilization modules can push the detection limit very low [31], ultimately limited by the absorption properties of the analyte [45]; (ii) almost linear operation in a broad range of RIU; (iii) compact dimensions limited by the size of the probe beam, planar geometry involving only one lithographic step, and free-space operation; (iv) geometrically scalable design to other target wavelengths; (v) all-dielectric configuration, avoiding ohmic losses as in plasmonics-based sensors; and (vi) potential for integration in microfluidic setups by simply confining the analyte volume with a planar superstratum. Finally, inverting the principle of operation, the same device has the potential to function as a highly selective tunable optical notch filter by substituting the analyte material with an electro-optically tunable medium, such as nematic liquid-crystals [50,51].

## 4. Conclusions

It has been demonstrated that the interference between the electric and toroidal dipole leading to anapole modes occurs and can be efficiently tuned in hollow silicon cuboid nanoparticles. Once the anapole mode is identified, a metasurface is designed based on the scattering properties of individual nanoparticles, which are investigated by the multipole decomposition method. It has been observed that hollow nanocuboids show several advantages over solid ones. The anapole mode can be excited for a broad range of refractive indices of the surrounding medium. Moreover, when arranged in a metasurface configuration, the holes lead to strong light confinement with associated very low radiative decay rates and hence resonances with ultrahigh quality factors. The anapole resonances are significantly tuned by variations of the superstratum index, enabling refractometric sensing of an analyte in contact with the metasurface. By optimizing the geometrical parameters, sub-picometric resonance linewidths are achieved, which is a critical property in the design of sensors with ultralow detection limits. In addition, being all-dielectric, the proposed sensors do not suffer from ohmic losses. All these factors state the potential of the proposed metasurfaces based on hollow dielectric nanoparticles for refractometric applications. Moreover, thanks to their optical spectral response and high *Q*-factors, such devices can be potentially exploited in other fields based on enhanced light–matter interaction, e.g., applications in non-linear devices, tunable switches, and optomechanics.
